# Isolation of a Novel QTL, *qSCM4,* Associated with Strong Culm Affects Lodging Resistance and Panicle Branch Number in Rice

**DOI:** 10.3390/ijms24010812

**Published:** 2023-01-03

**Authors:** Xianli Yang, Yongcai Lai, Lizhi Wang, Minghui Zhao, Jiayu Wang, Mingxian Li, Liyong Chi, Guoyi Lv, Youhong Liu, Zhibo Cui, Rui Li, Liren Wu, Bing Sun, Xijuan Zhang, Shukun Jiang

**Affiliations:** 1 Crop Cultivation and Tillage Institute, Heilongjiang Academy of Agricultural Sciences, Harbin 150086, China; yangxianli@haas.cn (X.Y.); laowantong@haas.cn (Y.L.); lizhiwang@haas.cn (L.W.); limingxian@haas.cn (M.L.); chliyong@haas.com (L.C.); guoyilv@haas.cn (G.L.); hongniu@haas.cn (Y.L.); lirui1@haas.cn (R.L.); longjiangjiangguo@haas.cn (L.W.); sunbing@haas.cn (B.S.); 2 Heilongjiang Provincial Key Laboratory, Crop Physiology and Ecology in Cold Region, Heilongjiang Provincial Engineering Technology Research Center of Crop Cold Damage, Harbin 150086, China; 3 Northeast Center of National Salt-Alkali Tolerant Rice Technology Innovation Center, Harbin 150086, China; 4 Rice Research Institute, Shenyang Agricultural University, Collaborative Innovation Center Co-Sponsored by Liaoning Provincial Government and Ministry of Education for Northeast Japonica Rice Genetic Improvement and High Efficiency Production, Shenyang 110161, China; mhzhao@syau.edu.cn (M.Z.); wangjiayu@syau.edu.cn (J.W.); cuizb@syau.edu.cn (Z.C.); 5 Qiqihar Branch, Heilongjiang Academy of Agricultural Sciences, Qiqihar 161006, China

**Keywords:** rice, lodging resistance, functional analysis, *qSCM4*, candidate gene

## Abstract

Rice breeders are now developing new varieties with semi-high or even high plant height to further increase the grain yield, and the problem of lodging has re-appeared. We identified a major quantitative trait locus (QTL), *qSCM4,* for resistance to lodging by using an F_2_ segregant population and a recombinant self-incompatible line population from the cross between Shennong265 (SN265) and Lijiangxintuanheigu (LTH) after multiple years and multiple environments. Then, the residual heterozygous derived segregant population which consisted of 1781 individual plants, and the BC_3_F_2_ segregant population which consisted of 3216 individual plants, were used to shorten the physical interval of *qSCM4* to 58.5 kb including 11 genes. DNA sequencing revealed the most likely candidate gene for *qSCM4* was *Os04g0615000*, which encoded a functional protein with structural domains of serine and cysteine. There were 13 DNA sequence changes in LTH compared to SN265 in this gene, including a fragment deletion, two base changes in the 3′ UTR region, six base changes in the exons, and four base changes in the introns. A near-isogenic line carrying *qSCM4* showed that it improved the lodging resistance through increasing stem thickness by 25.3% and increasing stem folding resistance by 20.3%. Furthermore, it was also discovered that *qSCM4* enhanced the primary branch per panicle by 16.7%, secondary branch by per panicle 9.9%, and grain number per panicle by 14.7%. All the above results will give us a valuable genetic resource for concurrently boosting culm strength and lodging resistance, and they will also provide a basis for further research on the lodging resistance mechanism of rice.

## 1. Introduction

Lodging is one of the major problems preventing the increase of rice yield [1]. In severe cases, yield could be reduced by more than 50% or even fail completely. Moreover, lodging can increase humidity in the canopy and within the population [2], and cause fungal diseases [3] and pre-harvest sprouting, all of which would affect the quality and appearance of rice [1,4]. It mostly happens in the middle and end rice filling stages [4]. Because the dry organic matter in the stem sheath is continuously transferred to the spikelet, the mechanical strength of the stem sheath weakens with the increase of grain, causing lodging [5]. Rice lodging is classified into three types: bending lodging, frustrated lodging, and torsional lodging [4]. The widespread use of the Green Revolution gene “*sd1*” effectively reduced the occurrence of lodging and significantly improved fertility resistance and the harvest index of rice. However, due to the low biomass accumulation of dwarf plants, the utilization rate of population light energy was reduced, which reduced the biological yield and limited the yield improvement [6,7]. As a result, in the 1990s, the use of strain improvement for ultra-high yield rice breeding and selection of super rice began to achieve yield improvement by increasing the yield of varieties [8,9,10]. However, increasing plant height inevitably led to an increase in biological yield, and the problem of lodging resurfaced.

The plant height, pushing resistance of the lower part, internode number, internode length, stem diameter, stem wall thickness, vascular bundles and other morphological characteristics all bear relation to rice lodging [11,12,13,14,15]. Researchers mined and cloned QTLs for a number of lodging-resistant features and conducted biological function investigations. The dwarf genes *sd1*, *d11*, *d14*, *d61*, and *SBI* played a role in controlling the synthesis and metabolism of hormones such as gibberellin (GA) and rapeseed lactone (BR), which primarily altered internode length and stem wall thickness and, consequently, the plant’s resistance to lodging [14,16,17,18]. The mutations in the cellulose synthases, which were encoded by *OsCESA4*(*Bc7*), *OsCESA7*, and *OsCESA9*(*Bc6*), led to the production of defective secondary wall cellulose and decreased stem strength [19,20,21,22]. The mutants of *OsBC17*, which were a novel allele of *TAC4*, have lower lignin content and weak lodging resistance [23]. Reduced cellulose content, thinner secondary cell walls, and decreased mechanical strength of culm were the results of mutations in the kinesin genes *OsDRP2B*(*Bc3*), *BC15*(*OsCTL1*), and *BC10* [24,25,26,27]. The *OsEXTL* gene transgenic plants showed remarkably thickened secondary cell walls with higher cellulose levels, resulting in a significant increase in detectable mechanical strength [28]. By boosting cellulose content, the bending-resistant lodging gene *BSUC11* prevented the physical strength of the upper culm from declining [29].

Furthermore, varieties carrying the ideal plant type gene *IPA1* frequently exhibited thick culm and a high resistance for lodging [30]. The *OsCAD2* gene for thick and stiff culm could improve the lodging resistance of lignin-deficient rice varieties [31]. Several known genes for strong culm resistance were involved in culm morphology regulation. *SCM1*(*Gn1a*), *SCM2*(*APO1*) and *SCM3*(*OsTB1*) improved culm structure and increased the plant lodging resistance by increasing stem diameter, stem wall thickness, and the number of mechanical tissue cells [32,33,34,35]. *SBI* increased the lodging resistance of rice by shortening the length of the basal internodes [17]. *Smos1* improved resistance to lodging of rice by increasing the number of cells in the stem and stem wall [36]. The *OsCKX2*/*Gn1a* gene not only affected culm development, but it was also expressed in root vascular tissues, and if it was not expressed, it would promote the growth of adventitious roots, which resulted in a more developed root system and improved plant resistance to lodging [37].

The discovery and functional analysis of genes associated with rice strong culm and lodging resistance provided components for rice breeding and subsequent breeding assisted by molecular markers, and led to further improvement in rice yield. Based on this, a major QTL for lodging resistance, named *qSCM4*, was identified via an F_2_ segregant population and a recombinant self-incompatible line population from the cross between Shennong265 (SN265) and Lijiangxintuanheigu (LTH) in multi years and multi environments. Then, the *qSCM4* was fine mapped into a 58.5 kb region in a residual heterozygous derived segregant population and a BC_3_F_2_ segregant population. DNA sequencing revealed the most likely candidate gene for *qSCM4* was *Os04g0615000*, which encoded a functional protein with serine and cysteine structural domains. The effects of *qSCM4* on the lodging and yield related traits also were analyzed by using the near-isogenic lines. The objectives of this research were to give a useful genetic resource for concurrently boosting culm strength and lodging resistance and provide a basis for further research on lodging resistance mechanism of rice.

## 2. Results

### 2.1. QTL Mapping of Yield and Lodging Related Traits on Chromosome 4 in Different Environments

**Figure 1:**
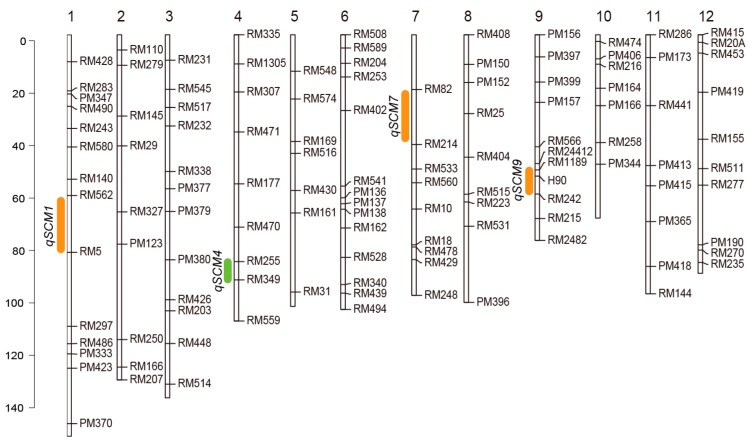
Genomic locations of four QTLs with strong effects for lodging related traits identified in the RILs population. Green indicates positive effect inherited from LTH. Orange indicates positive effect inherited from SN265. 1–12 indicates chromosome 1 to chromosome 12.

The two parents SN265 and LTH showed significant differences in number of grains per panicle (GPP), number of primary branches (PBN), number of secondary branches (SBN), breaking strength of the basal internode (BS), basal culm diameter (CD), and basal culm thickness of the basal culm (CT) under different environments (Table 1). We focused on breaking strength of the basal internode (BS) in the three lodging related traits. A total of four QTLs controlling BS were identified in chromosome 1, chromosome 4, chromosome 7, and chromosome 9 and named *qSCM1*, *qSCM4*, *qSCM7*, and *qSCM9*, respectively (Figure 1). Among these four QTLs, the alleles of *qSCM1*, *qSCM7*, and *qSCM9*, all had a positive effect inherited from SN265. Only *qSCM4* had a positive effect inherited from LTH. Furthermore, the QTLs controlling number of grains per panicle (GPP), number of primary branches (PBN), number of secondary branches (SBN), basal culm diameter (CD), and basal culm thickness of the basal culm (CT) were all detected in the region under different environments (Table 2). Further analysis revealed that no QTLs related to plant height and internode length were detected in this interval (Table 2), avoiding the effect of plant height on the analysis of lodging resistance at this locus.

### 2.2. Fine Mapping and Candidate Gene Analysis of the Strong Culm and Lodging Resistance QTL qSCM4

**Figure 2:**
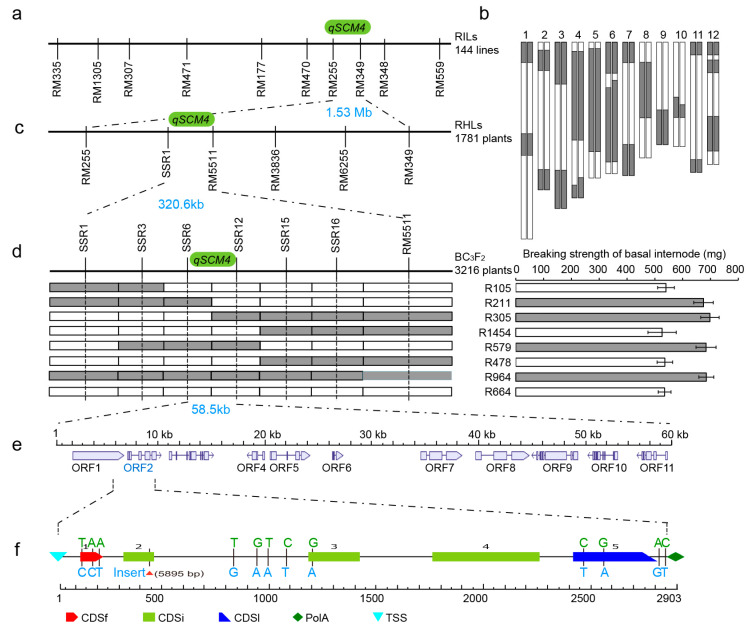
Fine mapping and candidate gene analysis of the strong culm and lodging resistance QTL *qSCM4*: (**a**) genetic localization of *qSCM4* in recombinant inbred lines population; (**b**) the genotypes of residual heterozygous line 08-3-2-1, The grey parts indicate the genotypes of the LTH, and the white indicate the genotypes of SH265; (**c**) fine mapping of *qSCM4* in remaining heterozygous-derived populations; (**d**) fine mapping of *qSCM4* in BC_3_F_2_ segregating populations, The grey parts indicate the genotypes of the LTH, and the white indicate the genotypes of SH265; (**e**) candidate gene prediction for *qSCM4* region; and (**f**) candidate gene DNA sequence variation of *qSCM4*.

The strong culm and lodging resistance QTL *qSCM4* was localized in the 1.53 Mb range by multi-year (Figure 2a, Table 2). A fine mapping of *qSCM4* was performed using a RHL derived segregating population containing 1781 single plants, whose progeny segregated as strong culms (1317) and weak culms (464) = 2.84:1.00 (χ^2^ = 0.4593, *p* > 0.05) conforming to a 3:1 segregation ratio. Combining the four newly-developed polymorphic molecular markers in the target region narrowed the localization of *qSCM4* from 1.53 Mb to 320.6 kb between SSR1-RM5511 (Figure 2c). Using the constructed BC_3_F_2_ backcrossing segregation populations containing 3216 individuals, the physical interval of *qSCM4* was finally narrowed to 58.5 kb by the screening of key recombinant individuals combined with phenotypic identification (Figure 2d).

The candidate gene annotation analysis of *qSCM4* in the rice genome using the RAP-DB database (http://rapdb.dna.affrc.go.jp/, accessed on 27 December 2022) revealed that the 58.5 kb range contained 11 annotated genes (Figure 2e). According to the annotation function of 11 genes and the re-sequencing date of the RIL parents (NCBI, PRJNA587802) [38]. Only *Os04g0615000*, which encoding a functional protein with serine and cysteine structural domains, was identified having biparental DNA sequence differences among these genes. There were 13 DNA sequence changes in LTH, compared to SN265 in this gene, including a fragment deletion, two base changes in the 3′ UTR region, six base changes in the exons, and four base changes in the introns. Four of these DNA sequence variations caused amino acid mutations including Val-Ala in exon 1, Arg-His in exon 3, Ala-Val and Val-Ile in exon 5, and two synonymous mutations on exon 1. The Arg-His mutation on exon 3 occurred at the active center of the functional structural domain and was likely to result in a change in protein function (Figure 2f).

### 2.3. Effect of qSCM4 on the Traits Related to Lodging and Panicle and Anatomical Structure Analysis

**Figure 3:**
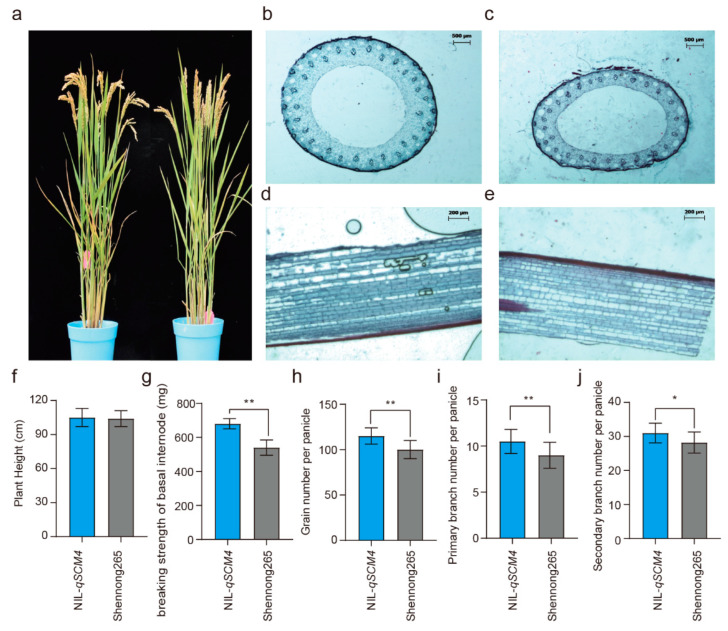
Analysis of yield and lodging-related traits in the near-isogenic lines with strong culm resistance QTL-*qSCM4*: (**a**) comparison of plant size of NILs; (**b**) cross section of the basal internode of NIL-*qSCM4*; (**c**) cross section of the basal internode stem of SN265; (**d**) the basal internode wall of NIL-*qSCM4*; (**e**) the basal internode stem wall of SN265; (**f**) comparison of plant height between two NILs; (**g**) comparison of breaking strength between two NILs; (**h**) comparison of grain number per panicle between two NILs; (**i**) comparison of the number of primary branch between two NILs; and (**j**) comparison of the number of secondary branch between two NILs. * *p* < 0.05, ** *p* < 0.01, using Student’s *t*-test.

Comparing the lodging resistance related traits and panicle yield traits of NILs revealed that NIL carrying *qSCM4* exhibited bigger basal culm diameter (Figure 3b,c), thicker basal culm thickness (Figure 3d,e), and larger breaking strength of the basal internode than SN265 (Figure 3g). Measurement of anatomical structural changes in the basal culm internode (Figure 3b–e) showed that NIL carrying *qSCM4* showed a 25.3% increase in basal culm diameter (Figure 3b,c), an average increase of 45.6% in the thickness of thin basal culm (Figure 3b,c) and an average increase of 13.6% in thick basal culm (Figure 3e). The plant type analysis of two NILs showed that NIL-*qSCM4* had no change in the plant type and height (Figure 3a,f). Analysis of yield-related traits showed that NIL-*qSCM4* had 14.7% more grains per panicle than SN265 (Figure 3h), 16.7% more primary branch (Figure 3i) and 9.9% more secondary branch (Figure 3j). The culm lodging resistance of NIL-*qSCM4* also increased by 20.3% (Figure 3g).

## 3. Discussion

Rice lodging is influenced by many factors in addition to plant height [4]. The intrinsic nature of rice lodging is that the basal culm is unable to support the weight of the upper plants. Therefore, the morphology and strength of the basal culm are also key factors in determining the rice lodging resistance. There is a highly significant positive correlation between basal culm thickness, basal culm diameter and culm strength [10,33,34,39,40]. The increasing of basal internode thickness and culm thickness could be an effective way to improve the rice lodging resistance [32]. Recent progress has shown that increasing basal culm thickness could significantly improve resistance to lodging. Furthermore, it has been demonstrated that only culm-diameter-related traits were positively correlated with lodging resistance, whereas plant height had an effect only within a certain range [15]. The main factor influencing culm wall thickness was the vascular bundle. The greater number of vascular bundles meant the more thicker culm wall and the greater resistance to bending and lodging [11]. *SCM3*/*OsTB1*/*FC1* was a key gene that regulated culm thickness, and overexpression of this gene could increase the culm thickness and improve the lodging resistance ability in rice [6,32]. The plant cell wall components including cellulose, hemicellulose, and lignin also influenced the lodging resistance ability [11]. In this study, *qSCM4* improved the lodging resistance through increasing basal culm thickness and culm wall thickness. The biological mechanism of *qSCM4* in maintaining culm mechanical strength and improving culm physical support requires further investigation.

The candidate gene in this study was identified as a novel allele of the well-known gene *NAL1* via the DNA sequence and variation analysis (Figure 2f). It was discovered that this gene could control leaf growth via influencing cell expansion and division [41]. *NAL1^Takanari^* had also been found to affect leaf photosynthesis by influencing leaf N, Rubisco, and chlorophyll content [42]. The allele *NAL1^Nipponbare^*/*LSCHL4* regulated flag leaf size, increased leaf chlorophyll content, the number of secondary branch and grains per panicle, and increased rice yield by coordinating source-sink relationships [43]. The effects of *NAL1^Lement^* allele were found involved in polar auxin/IAA transport [44]. The *NAL1^YP9^*/*SPIKE* allele regulated spike number, leaf size, root system and vascular bundle number [45]. Unlike the previous studies, we discovered that the *NAL1^LTH^*/*qSCM4* allele controlled the breaking strength of the basal internode (BS), basal culm diameter (CD), and basal culm thickness to improving the lodging resistance in rice.

Several important functional genes have been discovered to evince pleiotropy. *Ghd7* was found regulating not only heading stage, but also rice yield, plant height and leaf area [46]. The erect panicle gene *DEP1*, which was also be found controlling N-efficient, the number of grains per panicle, grain length and thousand grain weight [47]. The ideal strain gene *IPA1* has numerous functions that have been used in breeding, including increasing the number of primary branches, thickening culm, and enhancing plant disease resistance [48]. Future molecular breeding will have more resources if we can take advantage of the pleiotropy of genes. Rice breeding is presently advancing in the direction of molecular breeding as a useful technique for precise development. Researchers have bred new high-quality rice varieties after using molecular breeding techniques to improve varieties for real-world production requirements. In this study, the *qSCM4* was identified as creating effective molecular markers that could help us in the breeding of high-quality, high-yielding varieties with lodging resistance. The findings of this study offer a theoretical foundation for future investigation into the molecular regulatory mechanism regulating rice resistance to lodging and the creation of better varieties with high yields.

## 4. Materials and Methods

### 4.1. Mapping Population and Its Linkage Map

**Figure 4:**
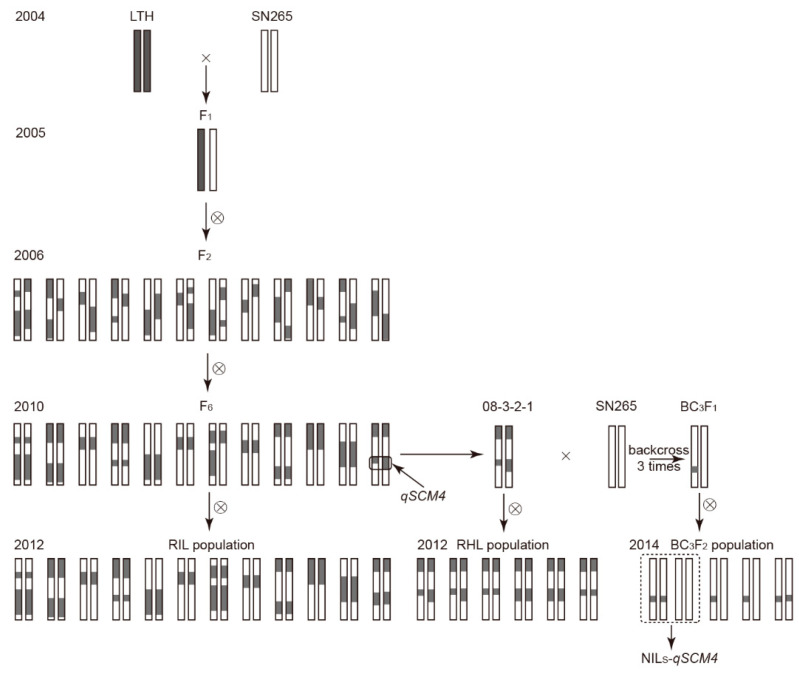
The building process of F_2_ population, RILs population, RHLs population and BC_3_F_2_ population used in this study.

Two rice varieties, ‘Shennong265’ (SN265) and ‘Lijiangxintuanheigu’ (LTH), having significant differences in lodging related traits, were utilized to produce several different-type mapping populations, as shown in Figure 4. In 2004, LTH was used as female to cross with SN265, and acquired the F_2_ population (176 plants) by self-fertilization in 2006. The single seed descent (SSD) method was used in sequent generations to build the recombinant self-incompatible lines (RILs) population from 2006 to 2010. In 2010, a residual heterozygous line (RHL) of *qSCM4*, which had heterozygous genotype in the *qSCM4* loci, was selected from the F_6_ generation population by using 114 SSR markers linkage map, and this line named 08-3-2-1. In 2012, the 144 RILs population and the RHLs population with 1781 plants were all achieved. From 2011 to 2014, a BC_3_F_2_ backcross segregating population with 3216 plants were generated using 08-3-2-1 as the female and SN265 as the parent through consecutive backcrosses and molecular marker tracking. A line named NIL-*qSCM4* with *qSCM4* region from LTH and genetic background of SN265 was selected from the BC_3_F_2_ population, and this line formed a pair of NILs with SN265 for studying the function of *qSCM4*.

### 4.2. Cultivation, Evaluation of Lodging and Yield Related Traits

The parents SN265 and LTH, F_2_ segregating population, and RILs segregating population, were all grown in the Rice Research Institute experimental fields of Shenyang Agricultural University (41°50′ N, 123°35′ E) in the rice growing seasons of 2004, 2006 and 2010 in Shenyang, Liaoning Province. The sowing date was 12 April, and 35-day-old seedlings of each plant (line) were transplanted at one seedling per hill on 20 May. The parents and each RIL line were planted in four rows of 15 hills at a spacing of 13.3 cm between hills and 30 cm between rows. Three replications were conducted according to a randomized block design. The F_2_ population plants were transplanted with 13.3 cm between plants and 30 cm between rows. Nitrogen (N), phosphorus (P), and potassium (K) fertilizers in the form of urea, calcium superphosphate, and potassium chloride were applied at rates of 120, 90, and 90 kg/ha, respectively. The other field management practices were done according to the most followed agricultural practices of local farmers.

The RHLs segregating population and BC_3_F_2_ backcross segregating population were planted with one seedling per hill in the Crop Cultivation and Tillage Institute experimental field of Heilongjiang Academy of Agricultural Sciences (45°50′ N, 126°50′ E), with a plant distance of 13.3 × 30 cm in 2012–2014 and 2016, respectively. The two NILs for *qSCM4* were planted in four rows of 10 hills per row in 2016. Nitrogen (N), phosphorus (P), and potassium (K) fertilizers in the form of urea, calcium superphosphate, and potassium chloride were applied at rates of 80, 60, and 60 kg/ha, respectively. The other field management practices were done according to the most followed agricultural practices of local farmers.

**Figure 5:**
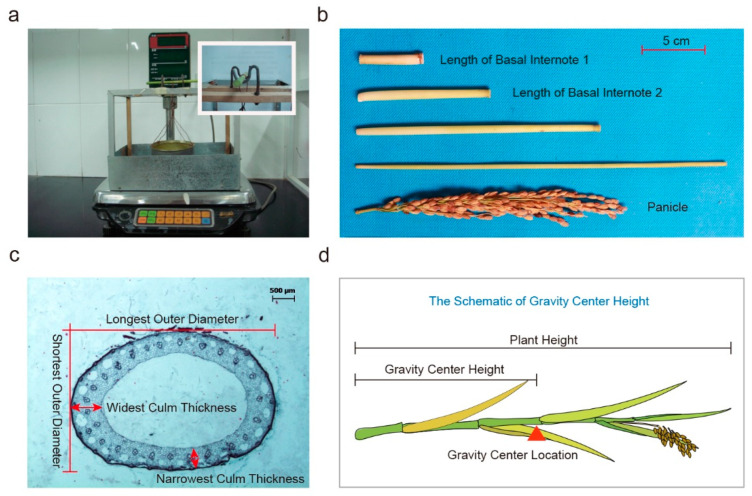
The measurement for lodging resistance related traits in rice: (**a**) the measured instrument for breaking strength of the basal internode; (**b**) the internode of a rice plant; (**c**) the diameter and thickness of a cross-section of the middle point of the basal culm; and (**d**) the schematic of gravity center height.

At 25 days after full heading stage, seven lodging related traits including breaking strength of the basal internode (BS) (Figure 5a), panicle fresh weight (PW), length of the lower two internode (LBI-1, LBI-2) (Figure 5b), basal culm diameter (the mean value of longest outer diameter and shortest out diameter) (CD), basal culm thickness of a cross-section of the middle point of the basal culm (CT) (Figure 5c), plant height (PH) and gravity center height (the distance from culm base to gravity center, GH) (Figure 5d) of 3–5 selected main stems from each single plant were measured. At mature period, 10 medium-length holes from each NIL were selected for trait investigation. Grain number per panicle (GPP), primary branch number per panicle (PBN), and secondary branch number per panicle (SBN) were all calculated individually.

### 4.3. QTL Analysis, Fine Mapping of qSCM4 and Candidate Gene Analysis

The CIM method of WinQTL Cartographer 2.5 was used for QTL localization, with the step rate set to 1 cM, LR threshold set to 11.5, model set to 6, and the number of control markers set to 5, and a control window of 10 cM on each side was selected for the examined interval using the forward regression method [49]. The LOD threshold (α = 0.05) was determined by the method of arranging the combinations 1000 times, and when the actual LOD value obtained was greater than the LOD threshold, it was considered that there was one QTL in the interval, and its confidence interval was the interval of LOD peak down by 1-LOD value. Fine mapping of *qSCM4* and selection of key recombinant plants were performed according to Fan’s report [50].

### 4.4. DNA Sequence of Os04g0615000

DNA sequence of the candidate gene was performed with reference to Jiang’s method [51]. Seven pairs of primer (Table 3) were designed in the *Os04g0615000* region to sequence the parents of the RILs by Biomarker Technologies (Beijing, China), in order to find differences.

### 4.5. Determination of Stalk Anatomical Structure

The 0.3–0.5 cm basal internode culms were sampled at 25 days after heading and fixed in FAA solution (38% formalin:acetic acid:70% alcohol = 1:1:18) for two days. Then, the samples were dehydrated in a graded series of ethanol concentrations after being de-silicified with 10% hydrofluoric acid for 25 d. The samples were infiltrated, embedded, and polymerized with the poly meth acryl resin, and were observed with a light microscope (ZeissA × 10) [32,34].

## 5. Conclusions

We identified a major quantitative trait locus (QTL), *qSCM4*, controlling the resistance to lodging, the number of grains per panicle, the number of primary and secondary branch, basal culm diameter, and basal culm wall thickness in multiple years and multiple environments. The physical interval of *qSCM4* was narrowed to 58.5 kb including 11 genes by using a residual heterozygous derived segregant population and a BC_3_F_2_ segregant population. DNA sequencing revealed the most likely candidate gene for *qSCM4* was *Os04g0615000*, which encoded a functional protein with serine and cysteine structural domains. There were 13 DNA variations between LTH and SN265 in this gene, including a fragment deletion, two base changes in the 3′ UTR region, six base changes in the exons, and four base changes in the introns. A near-isogenic line carrying *qSCM4* showed improved lodging resistance through increasing stem thickness by 25.3% and increasing stem folding resistance by 20.3%. Additionally, it was also discovered that *qSCM4* enhanced the primary branch per panicle by 16.7%, secondary branch per panicle by 9.9%, and the number of grains per panicle by 14.7%.

## Figures and Tables

**Table 1:** Distribution of several panicle characters and basal culm characters of SN265 and LTH in different environments.

Trait	Shenyang		Harbin	
	SN265	LTH	SN265	LTH
Number of grains per panicle (GPP)	162.07	126.43	161.07	128.32
Number of primary branches (PBN)	12.20	9.67	12.10	9.41
Number of secondary branches (SBN)	33.03	24.24	31.98	24.13
Breaking strength of the basal internode (BS)/g	883.09	366.50	879.25	367.22
Basal culm diameter (CD)/cm	4.54	4.10	4.38	4.06
Basal culm thickness of the basal culm (CT)/cm	1.40	0.58	1.38	0.59

**Table 2:** QTL for panicle and basal culm related traits on chromosome 4 using F_2_ population (176 plants) and RIL population (144 lines) of SN265-11/LTH in Shenyang and Harbin in 2008, 2010 and 2012, respectively.

Groups	Location	Trait	Year	Chr. ^a^	Interval	LOD ^b^	Add. ^c^	Var (%) ^d^
F2	Shenyang	BS	2008	4	RM470–RM559	4.80	−160	23.4
F2	Shenyang	CT	2008	4	RM470–RM559	3.19	−0.11	20.8
F2	Shenyang	CD	2008	4	RM470–RM559	3.34	−0.15	16.2
F2	Shenyang	GPP	2008	4	RM470–RM559	3.21	−6.1	16.8
F2	Shenyang	PBN	2008	4	RM470–RM559	5.90	−1.6	26.4
F2	Shenyang	SBN	2008	4	RM470–RM559	3.40	−2.5	16.1
RILs	Shenyang	BS	2010	4	RM225–RM349	5.21	−135	31.5
RILs	Shenyang	CT	2010	4	RM225–RM349	4.12	−0.18	24.5
RILs	Shenyang	CD	2010	4	RM225–RM349	4.36	−0.11	20.5
RILs	Shenyang	GPP	2010	4	RM225–RM349	3.21	−12.12	14.6
RILs	Shenyang	PBN	2010	4	RM225–RM349	13.34	−1. 1	43.2
RILs	Shenyang	SBN	2010	4	RM225–RM349	4.56	−5.47	26.6
RILs	Harbin	BS	2012	4	RM225–RM349	3.92	−157	28.7
RILs	Harbin	CT	2012	4	RM225–RM349	4.12	−0.18	24.5
RILs	Harbin	CD	2012	4	RM225–RM349	3.76	−0.14	22.7
RILs	Harbin	GPP	2012	4	RM225–RM349	3.37	−8.72	20.4
RILs	Harbin	PBN	2012	4	RM225–RM349	3.19	−0.98	19.4
RILs	Harbin	SBN	2012	4	RM470–RM559	3.56	−2.76	19.5

^a^ Chr., chromosome; ^b^ Logarithm (base 10) of the odds for the corresponding QTL peak; ^c^ Additive effect of the corresponding QTL; ^d^ Percentage of the phenotypic variation explained by the corresponding QTL.

**Table 3:** Seven pairs of primer information for DNA sequencing.

Marker	Forward Primer (5′–3′)	Reverse Primer (5′–3′)
Primers 1	CGCTTTCGGCATTCGTTATC	AGGTTGGATGTCGGCCATAA
Primers 2	CATAGCAAGATATTGCGGCGTTT	TGCTTAATGCACATGGTATTTTTGC
Primers 3	GGCTCAAACACCTAAAGAGCAAA	AACTCCTTCCAATCTCCGATCA
Primers 4	TCTTGTGCAGATTAAAGCTTCTGG	GGGTTTGTTCCAGCATAGATTCC
Primers 5	TGCAAAAAGTGCTGGTTCTGAATTT	TCCGGCAATGGTGTATATCAGGT
Primers 6	CATGGCCCTGAAAACTGGAC	GATGCCTCCTCCCCTTCAGT
Primers 7	CGCCATGTCGCTCCATCT	TCATGAGCACAGCAAAACTGC
